# Evaluating Cost‐Effective Strategies for Asymptomatic Microhematuria Diagnosis: A Risk‐Based Alternative to the American Urological Association Guidelines

**DOI:** 10.1002/jso.28148

**Published:** 2025-05-13

**Authors:** Krishay Sridalla, Hiten D. Patel, Dustin D. French, Joshua J. Meeks, Lili Zhao, Yuying Xing, David J. Bentrem

**Affiliations:** ^1^ Department of Surgery Northwestern University Feinberg School of Medicine Chicago Illinois USA; ^2^ Department of Urology Northwestern University Feinberg School of Medicine Chicago Illinois USA; ^3^ Department of Surgery Jesse Brown Veterans Administration Medical Center Chicago Illinois USA; ^4^ Department of Ophthalmology Northwestern University Feinberg School of Medicine Chicago Illinois USA; ^5^ Department of Veterans Affairs Center of Innovation for Complex Chronic Healthcare Hines Illinois USA; ^6^ Department of Medical Social Science Northwestern University Feinberg School of Medicine Chicago Illinois USA; ^7^ Department of Preventive Medicine (Biostatistics Division) Northwestern University Feinberg School of Medicine Chicago Illinois USA; ^8^ Corewell Health Research Institute Royal Oak Michigan USA

**Keywords:** bladder cancer, cost‐effectiveness, cystoscopy, microhematuria

## Abstract

**Background and Objectives:**

The American Urological Association (AUA) guidelines recommend evaluating asymptomatic microhematuria (MH) at ≥ 3 red blood cells per high powered field (RBCs/hpf), resulting in significant costs with limited bladder cancer detections. This study evaluates alternative diagnostic strategies to improve the cost‐effectiveness of asymptomatic MH evaluation.

**Methods:**

The cost‐effectiveness analysis compared three alternative strategies: Strategy 1 (cystoscopy at ≥ 26 RBCs/hpf) was compared to a 3 RBCs/hpf threshold, while Strategy 2 (cystoscopy and renal ultrasound at ≥ 3 RBCs/hpf) and Strategy 3 (cystoscopy and renal ultrasound at ≥ 26 RBCs/hpf) were compared to the AUA guidelines. Total costs, cost per patient evaluated, costs per cancer detected, and incremental cost‐effectiveness ratios (ICERs) were calculated.

**Results:**

Strategy 3 minimized costs without significantly reducing early cancer detection rates. It was cost‐effective for females (ICER = $120,649) and the total sample (ICER = $50,648) but not specifically for males (ICER = $23,326). Strategies 1 and 2 yielded lower cost savings and were less efficient.

**Conclusions:**

Strategy 3—performing cystoscopy and renal ultrasound for higher‐risk patients ( ≥ 26 RBCs/hpf)—offers a more cost‐effective approach than the AUA guidelines, particularly for women. Future studies should incorporate additional patient variables and diagnostic test characteristics.

**Abbreviations**
AUA: American Urological Association
BCa: bladder cancer
CMS: Centers for Medicare & Medicaid Services
CPT: current procedural terminology
CT: computed tomography
ICER: incremental cost‐effectiveness ratio
MH: microhematuria
OPPS: Hospital Outpatient Prospective Payment System
PFS: Physician Fee Schedule
QALY: quality‐adjusted life year
RBC/hpf: red blood cells per high‐powered field
WTP: willingness to pay

## 1 Introduction

Bladder cancer (BCa) places a significant burden on the American population. BCa is the fourth most common cancer in men and a common cancer in women, with more than 80,000 new cases and 17,000 new deaths each year in the United States [1]. BCa is also one of the most expensive cancers in the United States, with annual treatment costs estimated at $373 million [2]. These expenses encompass endoscopic procedures for surveillance, diagnostic imaging, intravesical therapies, systemic chemotherapy and immunotherapy, as well as surgical procedures. Non‐randomized, population‐based studies indicate that earlier detection of BCa can reduce BCa‐specific mortality [3], yet no major randomized trials have focused on preventative measures for BCa, making the stage at diagnosis the primary predictor of BCa‐related mortality [4]. Approximately 95% of patients with BCa present with hematuria, either microscopic (MH) or visible (gross hematuria) [5]. Urinalysis is the standard initial diagnostic tool, with further guidelines for evaluation differing between various US and international groups [6]. The 2020 American Urological Association (AUA) guidelines provide a risk‐based diagnostic strategy for MH: cystoscopy and renal ultrasound for lower‐risk (3–10 RBC/hpf) and intermediate‐risk (11–25 RBC/hpf) individuals, and cystoscopy with computed tomography (CT) urography for high‐risk cases (26+ RBC/hpf) [7]. Additionally, individuals over 60 years with a 30 pack‐year smoking history are classified as high‐risk under these guidelines.

While the AUA's approach has been predicted to detect the most cancers, it is also associated with the highest costs due to the large number of patients evaluated for MH who ultimately do not have BCa [8]. In fact, BCa detection rates among individuals with MH are notably low, ranging from 0.02% to 6.11% [9, 10], with significantly lower detection rates in individuals with 3–25 RBC/hpf compared to higher values [10]. MH itself is a common condition, with a prevalence of 2.4%–31.1% in healthy individuals [7]. Moreover, BCa detection rates are particularly lower in women and non‐smokers compared to men and smokers, respectively [10, 11, 12]. Together, these findings suggest that diagnostic procedures may be overutilized in detecting BCa, especially in lower‐risk demographics and lower RBC/hpf ranges.

Cystoscopy is the gold standard for detecting BCa in patients with MH, while renal ultrasound and CT urography are the preferred imaging modalities often performed alongside cystoscopy. While the AUA recommends CT urography for high‐risk cases (26+ RBC/hpf) [7], evidence suggests that CT urography can be safely replaced by renal ultrasound [13, 14]. Given that cystoscopy is the most sensitive method for detecting BCa [13], strategies combining cystoscopy with an imaging modality can be considered equivalent in terms of sensitivity [14]. With the radiation exposure and high costs associated with CT urography [14, 15, 16], these findings raise concerns that individuals may be undergoing CT urography unnecessarily, as renal ultrasound could provide a safer, cost‐efficient alternative without compromising diagnostic accuracy.

Therefore, we investigated the cost‐effectiveness of adjusting the AUA diagnostic strategy for MH. Using data from a retrospective population‐based cohort study by Jung et al. [10], we evaluated three alternative strategies designed to reduce the number of procedures performed while maintaining cancer detection rates. Overall, we sought to develop evidence‐based recommendations for more efficient evaluation of asymptomatic MH.

## 2 Materials and Methods

We conducted a cost‐effectiveness analysis using Microsoft Excel (Microsoft Corporation, Redmond, WA, USA) to compare three alternative guidelines for the initial diagnostic evaluation of MH [17]. Our analysis modeled outcomes and costs for a cohort of 105,704 adults with MH, using data from a retrospective population‐based cohort study by Jung et al., which reports the incidence of urinary tract cancers stratified by age, gender, and MH levels [10]. For this study, we included only patients aged 40 years and older and excluded those with 0–2 RBCs/hpf were excluded, as this range was beyond the scope of our investigation (Table 1). The data illustrates cancer incidence rates by RBC/hpf levels in males and females.

**Table 1:** Incidence of urinary tract cancers in males and females > 40 years by RBC/hpf [10].

RBC/hpf	Males		Females	
	Cancers/Total	% Cancer	Cancers/Total	% Cancer
3–10	280/23,350	1.20	106/49,076	0.22
11–25	88/4378	2.01	38/9414	0.40
26–99	193/4769	4.05	63/7241	0.87
100+	208/3407	6.11	72/4069	1.77

Abbreviation: RBC/hpf, red blood cells per high‐powered field.

We estimated the outcomes and costs for the cohort (*n* = 105,704) under three alternative diagnostic strategies, comparing each to a reference strategy. First, we compared the diagnostic thresholds of 3 RBC/hpf (*T* = 3) and 26 RBC/hpf (*T* = 26), also referred to as Strategy 1, for performing cystoscopy (Table 2). This comparison focused solely on cystoscopy thresholds without factoring imaging costs. We then expanded our analysis to include imaging procedures and compared the AUA guidelines with Strategy 2, which involves performing cystoscopy and renal ultrasound for all patients above 3 RBC/hpf (Table 3). Strategy 3 combines elements of Strategies 1 and 2 by recommending cystoscopy and renal ultrasound only for patients with RBC/hpf values above 26. For simplicity, we assumed in our analysis that under the AUA guidelines, all patients with 3–10 RBCs/hpf underwent cystoscopy and renal ultrasound, excluding the option of repeating urinalysis within 6 months.

**Table 2:** Evaluation algorithms for *T* = 3 RBC/hpf and *T* = 26 RBC/hpf (Strategy 1).

RBC/hpf	Diagnostic strategy	
	*T* = 3 RBC/hpf	*T* = 26 RBC/hpf (Strategy 1)
3–10	Cystoscopy	None
11–25	Cystoscopy	None
26+	Cystoscopy	Cystoscopy

Abbreviation: RBC/hpf, red blood cells per high‐powered field.

**Table 3:** Evaluation algorithms for AUA guidelines, Strategy 2, and Strategy 3.

RBC/hpf	Diagnostic strategy		
	AUA	Strategy 2	Strategy 3
3–10	Repeat Urinalysis within 6 months OR Cystoscopy and Renal Ultrasound	Cystoscopy and Renal Ultrasound	None
11–25	Cystoscopy and Renal Ultrasound	Cystoscopy and Renal Ultrasound	None
26+	Cystoscopy and CT Urogram	Cystoscopy and Renal Ultrasound	Cystoscopy and Renal Ultrasound

Abbreviations: AUA, American Urological Association; CT, computed tomography; RBC/hpf, red blood cells per high‐powered field.

The costs of procedures, including cystoscopy, renal ultrasound, and CT urography, were estimated from the perspective of the US national payer (Medicare) using 2023 US dollars. These cost estimates were derived from the Centers for Medicare & Medicaid Services (CMS) Physician Fee Schedule (PFS) and the CMS Hospital Outpatient Prospective Payment System (OPPS) for January 2023, which respectively provide the physician and hospital outpatient fees for each procedure (Table 4) [18, 19]. Current Procedural Terminology (CPT) codes were used to identify the costs associated with each procedure. Costs were estimated based on the initial diagnostic evaluation, excluding costs related to subsequent testing.

**Table 4:** Costs of procedures.

Procedure	CPT code	Physician fee ($)	Hospital outpatient fee ($)	Total fee ($)
Cystoscopy	52,000	244.33	625.34	869.67
Renal ultrasound	76,770	110.81	106.88	217.69
CT urography	74,178	361.24	368.43	729.67

Abbreviation: CPT, Current Procedural Terminology.

### 2.1 Cost‐Effectiveness Analysis

We compared the three alternative strategies to their respective reference strategies in terms of total costs, cost per patient evaluated, and cost per cancer detected. The total number of cancers detected initially corresponds to the sum of the number of cancers identified across all RBC/hpf intervals (Table 1). This represents an initial count, as it excludes cancers that may be diagnosed at a later time. The number of cancers detected per patient evaluated was determined by dividing the total number of cancers by the total number of patients. Total procedural costs were calculated by multiplying the unit cost of the procedure (Table 4) by the total number of patients, assuming all patients underwent the procedure. The cost per patient evaluated was derived by dividing the total procedural cost by the total number of patients, while the cost per cancer detected was derived by dividing the total procedural cost by the total number of cancers.

The effectiveness of diagnostic strategies was quantified using quality‐adjusted life years (QALYs), a standard metric in cost‐effectiveness analyses [20]. To convert from cancer detections to QALYs, we used a factor of 4, consistent with the range of 2–7 provided by Heijnsdijk et al. for non‐muscle invasive BCa [21]. The incremental cost‐effectiveness analysis was conducted by calculating the incremental cost‐effectiveness ratio (ICER) for each strategy, defined as the incremental cost divided by the incremental QALYs gained. Cost‐effectiveness was further assessed by plotting incremental costs against incremental effectiveness relative to a willingness to pay (WTP) threshold. A WTP threshold of $50,000/QALY was used, reflecting a widely accepted benchmark [22]. ICER analysis was not performed for Strategy 2 versus the AUA guidelines, as there was estimated to be no difference in effectiveness.

## 3 Results

### 3.1 Cost‐Effectiveness Analysis for Strategy 1: Cystoscopy for ≥ 26 RBC/hpf

**Figure 1:**
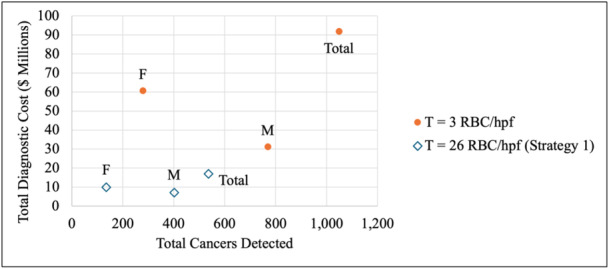
Total cancers detected initially and total diagnostic cost for *T* = 3 RBC/hpf and *T* = 26 RBC/hpf (Strategy 1). Abbreviations: F, females; M, males; RBC/hpf, red blood cells per high‐powered field.

When considering use of cystoscopy only, performing cystoscopy on all patients above the threshold of 3 RBC/hpf (Strategy 1) detected more cancers initially (1,048 vs. 536 cancers) but incurred higher costs ($91,927,598 vs. $16,946,390) when compared to performing cystoscopies on patients above the threshold of 26 RBC/hpf (*T* = 26) (Table 5, Figure 1). Notably, the difference in number of cancers initially detected between strategies was higher for males than for females (368 vs. 164 cancers).

**Table 5:** Total cancers detected initially and total diagnostic cost for *T* = 3 RBC/hpf and *T* = 26 RBC/hpf (Strategy 1).

Sex	*T* = 3 RBC/hpf		*T* = 26 RBC/hpf (Strategy 1)	
	Total cancers detected initially	Total diagnostic cost ($)	Total cancers detected initially	Total diagnostic cost ($)
Males	769	31,224,632	401	7,110,422
Females	279	60,702,966	135	9,835,968
Total	1,048	91,927,598	536	16,946,390

Abbreviation: RBC/hpf, red blood cells per high‐powered field.

**Figure 2:**
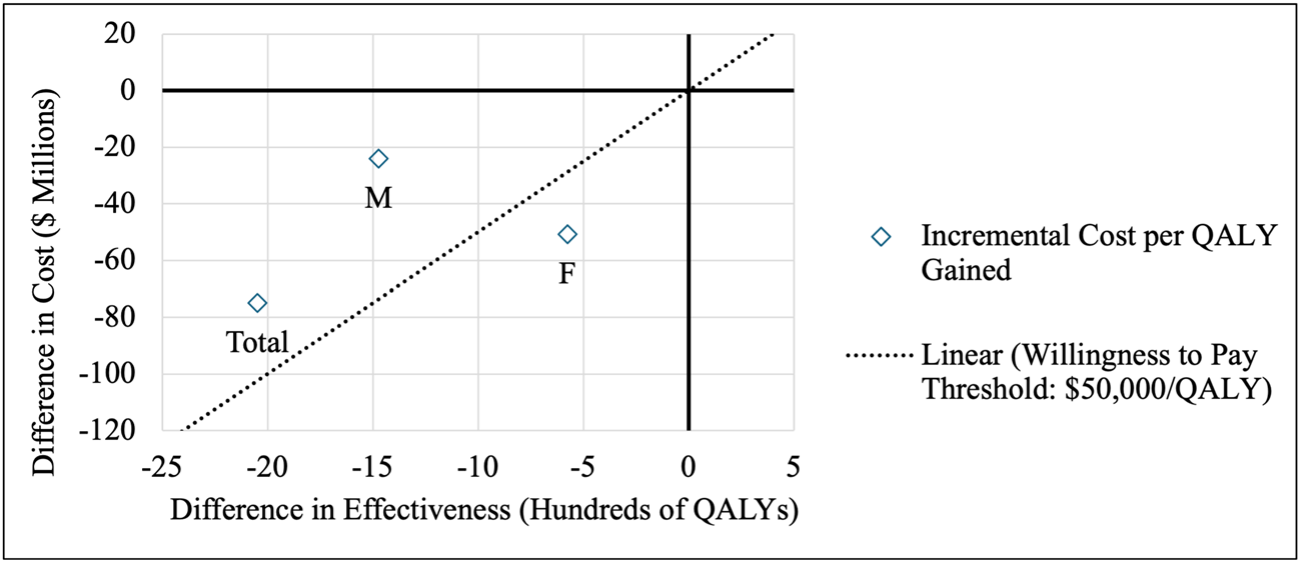
Incremental cost per QALY gained for *T* = 26 RBC/hpf (Strategy 1) in comparison to *T* = 3 RBC/hpf. Abbreviations: F, females; M, males; QALY, quality‐adjusted life year; RBC/hpf, red blood cells per high‐powered field.

The incremental cost‐effectiveness analysis revealed that incremental QALYs lost were higher for males (1472 QALYs) than for females (576 QALYs) but utilizing the 26 RBC/hpf threshold resulted in lower costs for females than males ($50,866,998 vs. $24,114,210) (Table 6). The ICER was highest for females ($88,311), followed by the overall sample ($36,612), and then males ($16,382). The 26 RBC/hpf threshold was cost‐effective for females, with the ICER falling to the right of the $50,000/QALY WTP threshold (Figure 2). However, the ICER did not meet the WTP threshold for males and the total population.

**Table 6:** Incremental cost per QALY gained for *T* = 26 RBC/hpf (Strategy 1) in comparison to *T* = 3 RBC/hpf.

Strategy	Sex	QALYs		Cost ($)		ICER
		Total	Incremental	Total	Incremental	
*T* = 3 RBC/hpf*	Males	3076	—	31,224,632	—	—
	Females	1116	—	60,702,966	—	—
	Total	4192	—	91,927,598	—	—
T = 26 RBC/hpf (Strategy 1)	Males	1604	−1472	7,110,422	−24,114,210	16,382
	Females	540	−576	9,835,968	−50,866,998	88,311
	Total	2144	−2048	16,946,390	−74,981,208	36,612

Abbreviations: ICER, incremental cost‐effectiveness ratio; QALY, quality‐adjusted life year; RBC/hpf, red blood cells per high‐powered field.

* *T* = 3 RBC/hpf was used as the reference strategy.

### 3.2 Cost‐Effectiveness Analysis for Strategy 2: Cystoscopy and Ultrasound for ≥ 3 RBC/hpf

**Figure 3:**
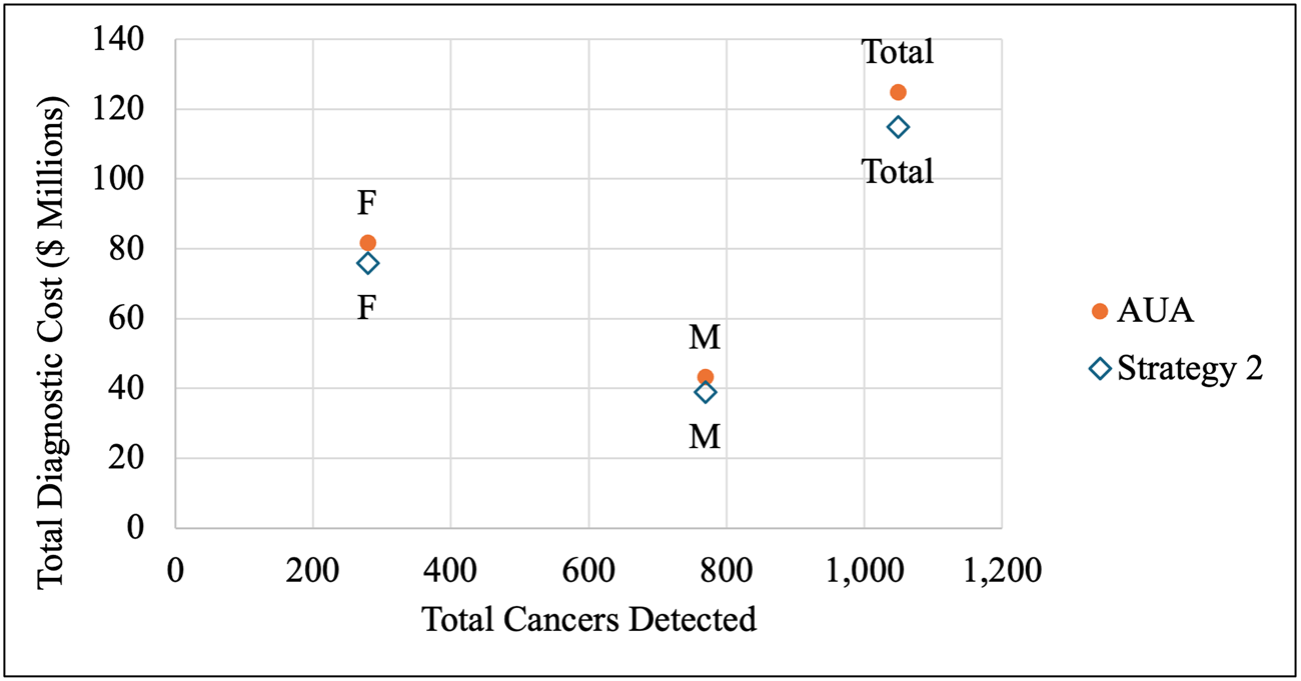
Total cancers detected initially and total diagnostic cost for AUA guidelines and Strategy 2. Abbreviations: AUA, American Urological Association; F, females; M, males.

**Figure 4:**
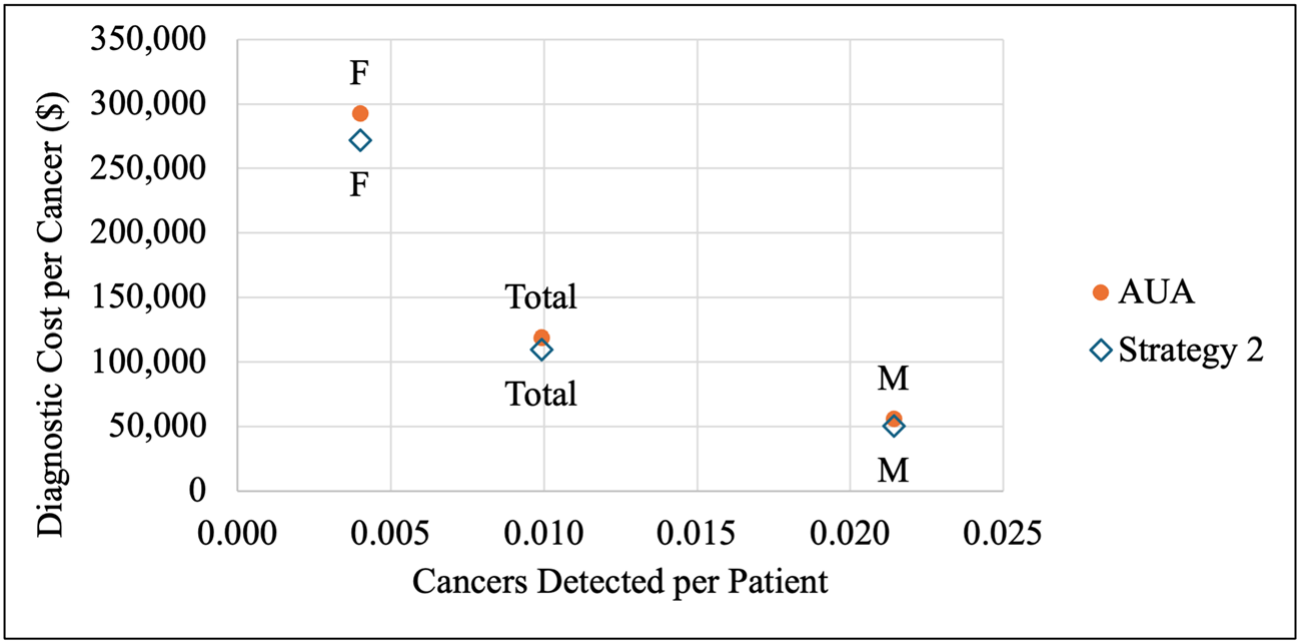
Cancers detected per patient evaluated and diagnostic cost per cancer for AUA guidelines and Strategy 2. Abbreviations: AUA, American Urological Association; F, females; M, males.

The AUA strategy incurred slightly higher total costs ($124,914,744 vs. $114,938,301), cost per patient evaluated ($1182 vs. $1087), and costs per cancer ($119,173 vs. $109,674) while detecting the same total cancers (1,048 cancers) and cancers per patient evaluated (0.0099) as Strategy 2 (Tables 7, 8; Figures 3, 4). When analyzing by sex, males demonstrated higher total cancer detections and cancer detections per patient evaluated and incurred lower total costs and costs per cancer than females for both strategies. However, diagnostic cost per patient evaluated were similar. The incremental cost‐effectiveness analysis revealed that adopting Strategy 2 resulted in a total cost savings of $9,976,442, with greater cost savings for females compared to males ($5,790,494 vs. $4,185,948). Both strategies yielded identical effectiveness (4192 QALYs), with no incremental QALYs observed.

**Table 7:** Total cancers detected initially and total diagnostic cost for AUA guidelines and Strategy 2.

Sex	AUA		Strategy 2	
	Total cancers detected initially	Total diagnostic cost ($)	Total cancers detected initially	Total diagnostic cost ($)
Males	769	43,226,522	769	39,040,573
Females	279	81,688,222	279	75,897,728
Total	1048	124,914,744	1048	114,938,301

Abbreviation: AUA, American Urological Association.

**Table 8:** Cancers detected per patient evaluated and diagnostic cost per cancer for AUA guidelines and Strategy 2.

Sex	AUA		Strategy 2	
	Cancers detected per patient evaluated	Diagnostic cost per cancer ($)	Cancers detected per patient evaluated	Diagnostic cost per cancer ($)
Males	0.0214	56,211	0.0214	50,768
Females	0.0040	292,789	0.0040	272,035
Total	0.0099	119,193	0.0099	109,674

Abbreviation: AUA, American Urological Association.

### 3.3 Cost‐Effectiveness Analysis for Strategy 3: Cystoscopy and Ultrasound for ≥ 26 RBC/hpf

**Figure 5:**
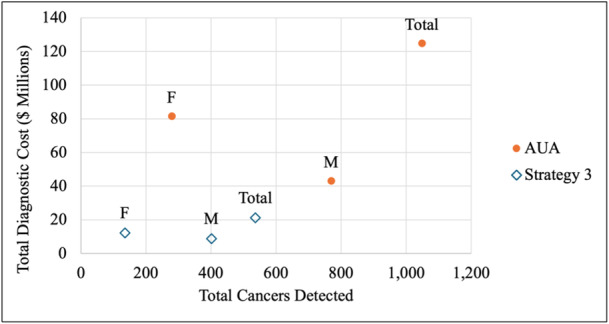
Total cancers detected initially and total diagnostic cost for AUA guidelines and Strategy 3. Abbreviations: AUA, American Urological Association; F, females; M, males.

**Figure 6:**
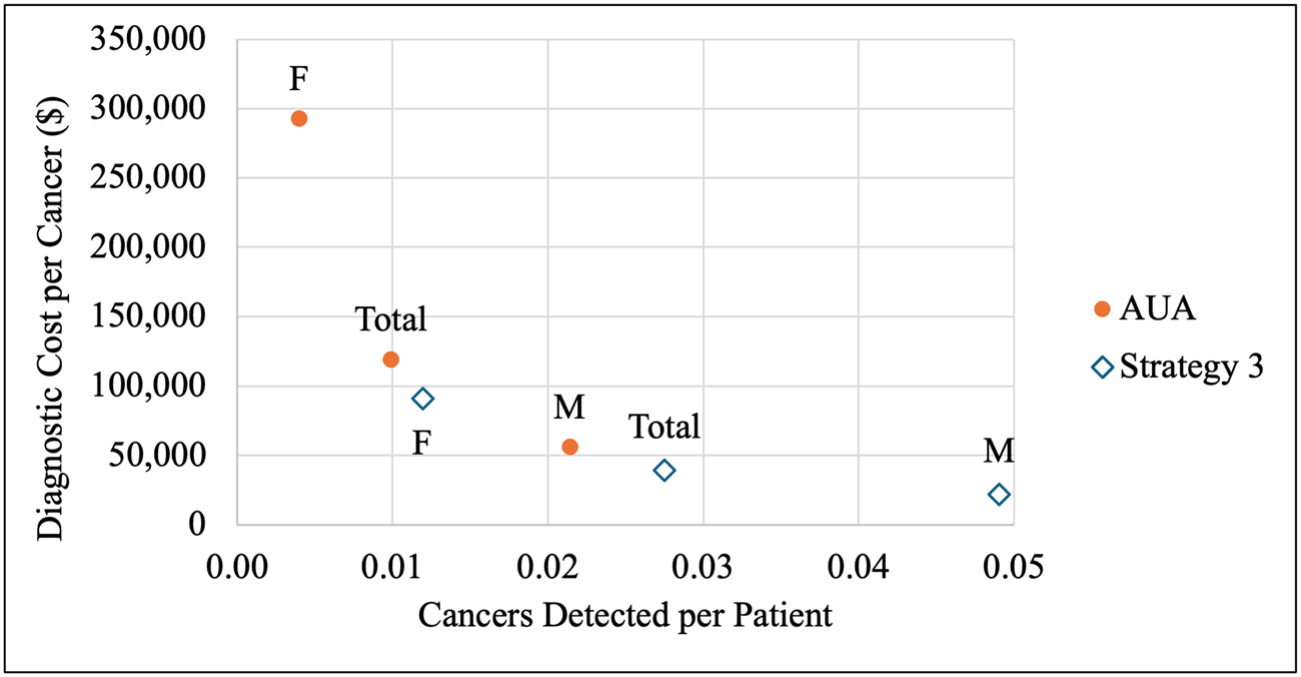
Cancers detected per patient evaluated and diagnostic cost per cancer for AUA guidelines and Strategy 3. Abbreviations: AUA, American Urological Association; F, females; M, males.

**Figure 7:**
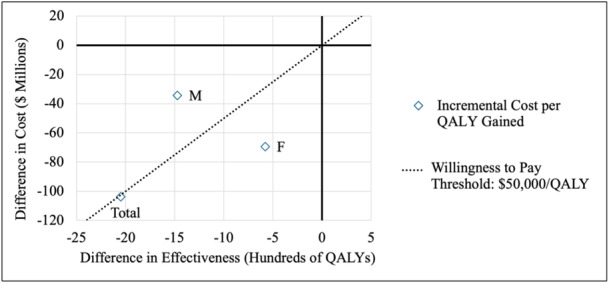
Incremental cost per QALY gained for Strategy 3 in comparison to AUA guidelines. Abbreviations: AUA, American Urological Association; F, females; M, males; QALY, quality‐adjusted life year.

The AUA guidelines detected higher total cancers (1048 vs. 536 cancers) and lower cancers per patient evaluated (0.0099 vs. 0.0275) but incurred higher total costs ($124,914,744 vs. $21,188,297), cost per patient evaluated ($1182 vs. $1087), and costs per cancer ($119,193 vs. $39,530) (Tables 9, 10; Figures 5, 6, 7). When evaluating by sex, males demonstrated higher total cancer detections and cancer detections per patient evaluated and incurred lower total costs, similar cost per patient evaluated, and higher costs per cancer compared to females. When comparing Strategy 3 with the AUA guidelines, males experienced a greater decrease in cancer detections (368 for males vs. 144 for females) and a smaller decrease in diagnostic costs ($34,336,267 for males vs. $69,390,180 for females) compared to females.

**Table 9:** Total cancers detected initially and total diagnostic cost for AUA guidelines and Strategy 3.

Sex	AUA		Strategy 3	
	Total cancers detected initially	Total diagnostic cost ($)	Total cancers detected initially	Total diagnostic cost ($)
Males	769	43,226,522	401	8,890,255
Females	279	81,688,222	135	12,298,042
Total	1048	124,914,744	536	21,188,297

Abbreviation: AUA, American Urological Association.

**Table 10:** Cancers detected per patient evaluated and diagnostic cost per cancer for AUA guidelines and Strategy 3.

Sex	AUA		Strategy 3	
	Cancers detected per patient evaluated	Diagnostic cost per cancer ($)	Cancers detected per patient evaluated	Diagnostic cost per cancer ($)
Males	0.0214	56,211	0.0490	22,170
Females	0.0040	292,789	0.0119	91,097
Total	0.0099	119,193	0.0275	39,530

Abbreviation: AUA, American Urological Association.

The incremental cost‐effectiveness analysis revealed that adopting Strategy 3 resulted in a total cost savings of $103,726,447. Similar to the analyses for Strategies 1 and 2, adopting Strategy 3 resulted in greater cost savings for females compared to males ($69,390,180 vs. $34,336,267) (Table 11). Incremental QALYs lost were higher for males (1472 QALYs) than for females (576 QALYs). The ICER was highest for females ($120,469), followed by the overall sample ($50,648), and then males ($23,326). Strategy 3 was cost‐effective for females and the overall population, with ICERs falling to the right of the $50,000/QALY WTP threshold (Figure 7). However, Strategy 3 was not cost‐effective for males.

**Table 11:** Incremental cost per QALY gained for Strategy 3 in comparison to AUA guidelines.

Strategy	Sex	QALYs		Cost ($)		ICER
		Total	Incremental	Total	Incremental	
AUA*	Males	3076	—	43,226,522	—	—
	Females	1116	—	81,688,222	—	—
	Total	4192	—	124,914,744	—	—
Strategy 3	Males	1604	–1472	8,890,255	–34,336,267	23,326
	Females	540	–576	12,298,042	–69,390,180	120,469
	Total	2144	–2048	21,188,297	–103,726,447	50,648

Abbreviations: AUA, American Urological Association; ICER, incremental cost‐effectiveness ratio; QALY, quality‐adjusted life year.

* The AUA guidelines were used as the reference strategy.

## 4 Discussion

Hematuria imposes a significant burden on the United States as it is one of the most common diagnoses treated by urologists, representing over 20% of all visits [7]. Compared to other US and international groups, the current AUA diagnostic strategy is associated with the highest costs due to the large number of individuals with MH who ultimately do not have BCa [8], which is exacerbated by the high prevalence of MH [7]. In this study, we investigated the cost‐effectiveness of three alternative strategies, focusing on potential improvements in efficiency and cost reduction.

We first evaluated the cost‐effectiveness of adjusting the cystoscopy threshold without factoring in imaging costs. Increasing the cystoscopy threshold from 3 to 26 RBC/hpf in Strategy 1 resulted in substantially lower total costs and a slight reduction in cancer detection, particularly among females. This aligns with prior findings that lower diagnostic thresholds lead to increased utilization of cystoscopy, and as a result, may be less cost‐effective in populations with lower disease prevalence [6, 10, 21, 23]. The ICER findings further demonstrated that the higher threshold is cost‐effective for females but not for males. Although these cost reductions come with a trade‐off in lower initial cancer diagnoses, it is important to consider that this represents an initial count. Patients who are not diagnosed under the new threshold would require repeat urinalysis, allowing for subsequent detection at later stages if and when RBCs for MH increased or gross hematuria developed, which could further improve the ICER and favor the higher RBC/hpf threshold.

While adjusting the cystoscopy threshold was cost‐effective, we also sought to independently assess the impact of changing the imaging modality from CT to ultrasound. Strategy 2 achieved slightly lower total costs, cost per patient evaluated, and costs per cancer than the AUA strategy, while maintaining equivalent cancer detection rates. These results align with Halpern et al.'s findings which demonstrate that replacing CT with cystoscopy can substantially reduce costs with minimal impact on cancer detection [14]. While our analysis did not address other drawbacks of CT, such as radiation exposure, contrast sensitivity, and the risk of secondary cancers highlighted by Georgieva et al. [6], these issues would also be mitigated by switching from CT to ultrasound. However, the cost reductions of switching to ultrasound were modest, which ultimately informed the development of Strategy 3.

Strategy 3, which combines elements of Strategies 1 and 2, incurred markedly lower total costs, cost per patient evaluated, and costs per cancer compared to the AUA guidelines, while moderately reducing total cancers detected and substantially increasing cancers detected per patient evaluated. The ICER plot further showed that Strategy 3 is cost‐effective not only for females but also for the total population, which indicates the substantial costs saved more than offset the lack of cancer detection at lower RBC/hpf values. However, Strategy 3 was not cost‐effective for males, emphasizing the need for sex‐specific diagnostic strategies given the differences in cancer detection rates [10, 12].

While Strategy 3 demonstrated significant cost savings, further advancements in diagnostic testing could refine MH management even further. Newer molecular tests may play a key role in optimizing diagnostic strategies by offering more precise diagnostic capabilities [24, 25], potentially influencing the RBC threshold for cystoscopy or even reducing the need for imaging in some cases. By providing both high diagnostic performance and cost‐effectiveness, molecular tests could help refine decision‐making processes in hematuria management. As these technologies evolve, their impact on diagnostic thresholds and imaging requirements will need to be considered, alongside the strategies discussed here, to improve both efficiency and patient outcomes.

This study has several limitations. Due to data constraints [10], we focused solely on RBC/hpf ranges in urine in males and females, excluding other AUA diagnostic criteria like smoking history, other BCa risk factors, history of gross hematuria, and previous urinalysis results [7], which could affect BCa detection rates and cost‐effectiveness [11, 26, 27, 28]. Additionally, the QALY conversion factor was based on data for patients with recurrent non‐muscle invasive BCa [21]. Since this estimate is based on recurrence, it may be conservative when applied to initial diagnosis, as some newly diagnosed cases may be cured without recurrence. However, we used this value to provide a cautious estimate of cost‐effectiveness. Also, the study population consisted of patients aged 65 years and older, while our cohort included individuals aged 40 and older, potentially limiting accuracy and generalizability [10]. Moreover, the higher proportion of females introduced a sampling bias, likely overestimating total diagnostic costs for females and influencing sex‐specific differences [10, 12], though this is unlikely to have significantly impacted the conclusions drawn within each sex. Additionally, our analysis assumed all cystoscopies were performed on an outpatient basis. We also assumed identical diagnostic efficacy between renal ultrasound and CT urography, which likely had minimal impact, as evidence suggests renal ultrasound is a safe alternative to CT [13]. Lastly, the analysis did not consider non‐cost‐related consequences of CT imaging, such as radiation exposure and contrast dye sensitivity [15, 29].

Future research should address the limitations of this study by incorporating a broader range of patient variables, including smoking history, prior gross hematuria, and additional BCa risk factors for better comparison to AUA guidelines [7]. Strategies should also be evaluated against other US and international guidelines, considering the variation in diagnostic approaches [6]. Expanding the sample size and age range would improve generalizability, and incorporating factors such as radiation exposure, contrast sensitivity, and risks of secondary cancers could enhance the evaluation of diagnostic strategies.

## 5 Conclusions

This study demonstrates a cost‐effective alternative to the AUA guidelines by recommending cystoscopies and renal ultrasounds for individuals with an RBC/hpf value over 25. Strategy 3 provides a more targeted and efficient approach to diagnosing MH, particularly for lower‐risk populations such as women, thereby supporting a risk‐based diagnostic strategy. These findings could inform future implementation efforts and prospective trial design to evaluate modifications to MH screening strategies. Further research could consider additional patient variables to further refine and validate the findings in other clinical contexts.

## Synopsis

Asymptomatic microhematuria is a common finding, and its diagnostic evaluation carries a substantial financial burden. This study estimated the costs and bladder cancer detection rates of various diagnostic strategies to determine a more cost‐effective approach. Raising the threshold for diagnostic testing and changing imaging modalities improved cost‐effectiveness compared to current guidelines.

## Data Availability

The data that support the findings of this study are publicly available and sourced from a previously published article (https://doi.org/10.1016/j.juro.2010.12.093). Our analyses are available from the corresponding author upon reasonable request.
